# Impact of the SARS-CoV-2 on the journey of high-risk colon cancer patients within the scope of the Unified Health System in Brazil

**DOI:** 10.1186/s12913-023-10083-9

**Published:** 2023-10-16

**Authors:** Raquel Pucci de Oliveira, Pedro Henrique Rezende de Moraes, Ana Paula Drummond-Lage

**Affiliations:** Faculdade Ciências Médicas de Minas Gerais, Alameda Ezequiel Dias 275, Belo Horizonte, 30.130.110 Brazil

**Keywords:** Chemotherapy (adjuvant, Metastatic), colon cancer, COVID-19, Delay, Pandemic, Sars-Cov-2

## Abstract

**Background:**

Colon cancer is an important cause of mortality related to cancer. During the COVID-19 pandemic, an important reallotment of assistance resources was necessary to tackle the crisis, directly impacting medical practice all over the globe.

**Objective:**

To assess the impact of the Sars-Cov-2 pandemic on the time between diagnosis and the beginning of systemic treatment in patients diagnosed with high-risk colon neoplasia.

**Methods:**

This is a retrospective study based on the analysis of medical records of patients diagnosed with colon neoplasia who required systemic treatment and were treated between March 2019 and March 2022, in a reference Oncology unit of the Brazilian Unified Health System. The study’s population was divided into two groups: (I) Pre-COVID-19: diagnoses made between March 2019 and February 2020, (II) COVID-19: diagnoses made between March 2020 and March 2022.

**Results:**

The sample consisted of 228 patients, 108 (47.97%) of whom were diagnosed during pre-COVID-19 and 118 (52.21%) diagnosed during the two years-period of COVID-19. Regarding the time between colonoscopy and surgery, the time between surgery and first consultation in clinical oncology, and the time between requesting and beginning of systemic treatment, a statistically significant reduction was observed during the COVID-19 period.

**Conclusion:**

A decrease in time between diagnosis and systemic treatment of patients with colorectal cancer during the COVID-19 pandemic was observed. Yet, even with this improvement, the time to begin treatment remains greater than the recommended by the current guidelines, regardless of the time of diagnosis (before or after the pandemic), which negatively impacts the disease outcome.

## Highlights

There was a decrease in colon cancer diagnoses during the COVID-19 period.

The number of stage III diseases increased during the COVID-19 period.

The time between diagnosis and treatment decreased during the COVID-19 period.

The beginning of systemic treatment was delayed compared to guidelines.

## Background

In December 2019 in Wuhan, China, the first cases of the disease caused by Sars-Cov-2 were identified (severe acute respiratory syndrome coronavirus 2 - Sars-CoV-2), namely COVID-19 [1]. The infection spread to the rest of the world, being declared a pandemic on March 11, 2020, by the World Health Organization (WHO) [2].

In Brazil, the first case of COVID-19 was diagnosed on February 26 2020 [3]. Due to the fast dissemination of the virus, an important reallotment of financial and assistance resources was necessary to tackle the crisis, directly impacting medical practice all over the world [4].

Due to the need to prioritize beds and other resources for patients contaminated by Sars-Cov-2, the health system became overwhelmed, resulting in the cancellation of cancer-tracking exams, as well as consultations and elective surgeries, disrupting oncological treatments [5].

Another factor that contributed to the delay in oncological care was the scientific evidence suggesting that patients undergoing antineoplastic treatment could develop more severe degrees of infection by COVID-19 [6].

In this scenario, this study aimed to assess the impact of COVID-19 on a highly prevalent malignant tumor. Colorectal cancer (CRC) is a leading cause of mortality related to cancer and, because of its high occurrence, it poses a problem to public health around the world. According to data from Global Cancer Statistics (GLOBOCAN), CRC is the third isolated cause of new diagnoses, representing 10% of new cases, and the second cause of cancer-related death [7].

Due to anatomic divergences, primary rectal and colon cancer require different staging procedures, surgical approaches, and treatments [8]. Furthermore, a systematic review identified that studies investigating the association between treatment intervals and survival are heterogeneous concerning treatment interval definitions, treatment interval time intervals, and used outcome measures for both tumors [9]. Considering this diversity, this study has focused only on colon cancer.

In addition to the staging, the time to begin treatment is a determining factor for outcomes, such as global survival rate and disease-free survival [10]. As a result, this study aimed to assess the impact of the Sars-Cov-2 pandemic on the time between diagnosis and the beginning of systemic treatment in patients diagnosed with high-risk colon neoplasia in the Brazilian Unified Health System (SUS). On top of that, it is the first Brazilian study with this goal, considering the SUS population.

## Methods

### Study design

This is a retrospective and observational study based on the analysis of medical records of patients diagnosed with colon neoplasia who requested systemic treatment and were treated between March 2019 and March 2022, in a reference oncology unit of the Unified Health System in Belo Horizonte, Brazil.

### Study population

This study’s population consisted of patients with colon neoplasia, including high-risk Clinical Stages (CS) II, III, and IV, who required systemic treatment.

**Fig. 1:**
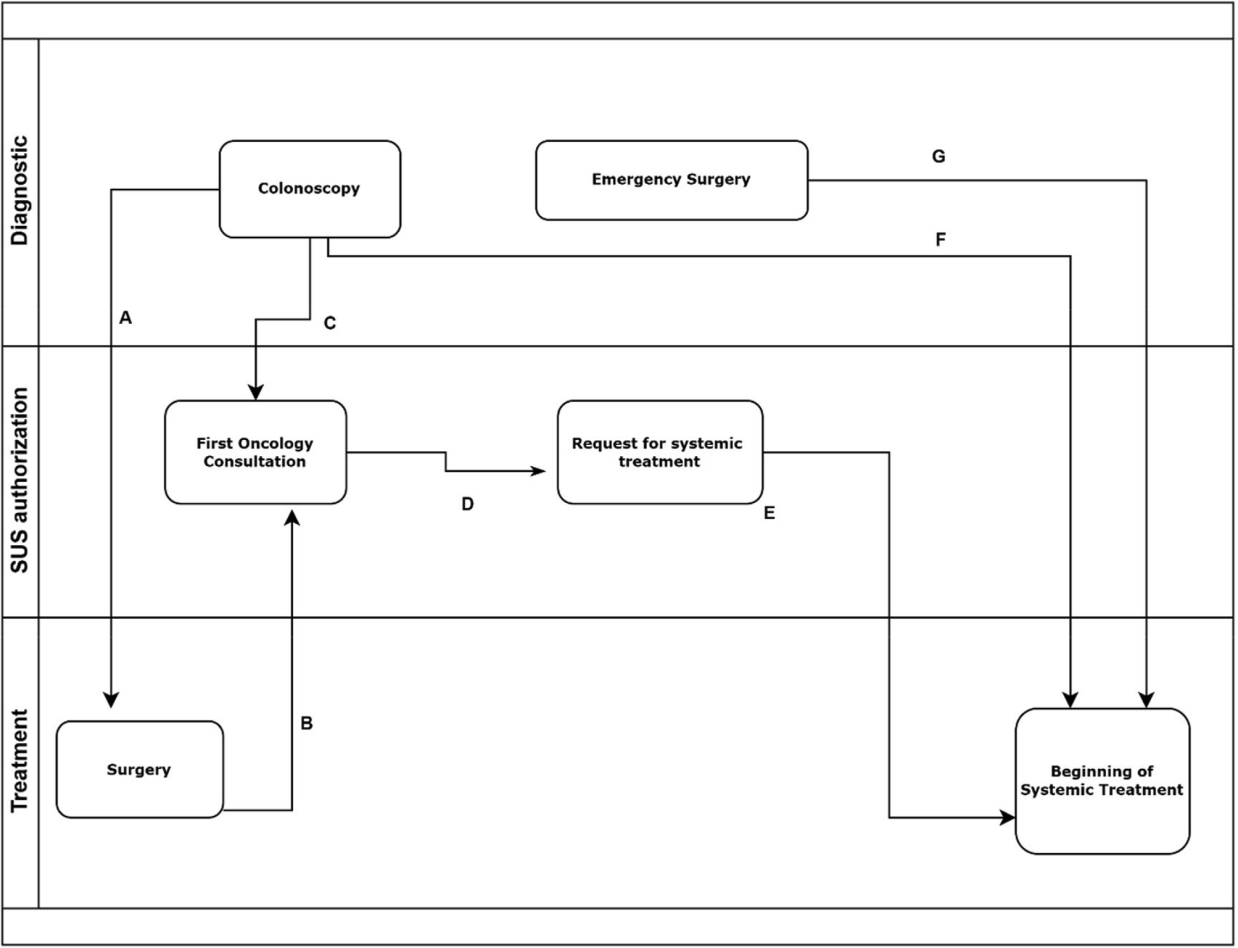
Flow of the oncological patient in the Unified Health System (SUS). (**A**) Time between diagnostic colonoscopy and surgery; (**B**) Time between surgery and the first oncological consultation; (**C**) Time between diagnostic colonoscopy and the first oncological consultation; (**D**) Time between the first oncological consultation and the request to systemic treatment; (**E**) Time between the application to systemic treatment and request of treatment; (**F**) Total time between diagnostic colonoscopy and beginning of systemic treatment; (**G**) Total time between diagnostic surgery and beginning of systemic treatment

Collected variables:

- Sociodemographic: age, sex, skin color, education, place of residence, time, and location of diagnosis.
- Clinical: performance status (PS), presence of comorbidities, alcoholism, smoking.
- Related to colon cancer: tumor location, method of diagnosis, type of surgery, presence of ostomy, type of treatment (adjuvant or metastatic), length of stay, after-surgery complications, and admittance to intensive care unit (ICU).
- Flow of patient care, in days, stratified, according to Fig. 1:

- Time between diagnostic colonoscopy and surgery;
- Time between surgery and the first oncological consultation;
- Time between diagnostic colonoscopy and the first oncological consultation (specific for patients with CS IV who weren’t submitted to surgery);
- Time between the first oncological consultation and the request for systemic treatment;
- Time between the request for systemic treatment and beginning of treatment;
- Total time between diagnostic colonoscopy and beginning of systemic treatment;
- Total time between diagnostic surgery and beginning of systemic treatment.

The study’s population was divided into two groups:

- Pre-pandemic (pre-COVID-19): diagnoses made between March 11, 2019, and March 10, 2020.
- During the pandemic (COVID-19): diagnoses made between March 11, 2020, and March 10, 2022.

The period defined as pandemic considered the date 03/11/2020 when the WHO classified the state of contamination pandemic [2].

The data were presented in frequency tables including their absolute frequencies and their respective percentages, as well as the descriptive measures (median and standard deviation) for quantitative data. The Kolmogorov-Smirnov test was used to assess normality. For parametric variables, the t-test was used to compare two medians, while the Mann-Whitney and Kruskal-Wallis test was used for non-parametric median comparison. The categorical variables were compared through Pearson’s chi-squared test; the Fisher test was used for expected frequencies smaller than 5, and Monte Carlo simulation was used in cases of more than two categories of response. The adopted significance level was 5% for all tests. The software utilized for analysis was the SPSS version 25.0.

## Results

The number of patients with a new diagnosis of colon cancer between March 2019 and March 2022 was 228, of which 108 (52.21%) patients were diagnosed in the pre-COVID-19 period and 118 in the COVID-19 period. As the COVID-19 period consisted of two years, the average number of diagnoses per year was 59 cases, meaning a 45% reduction.

There was no significant difference between the groups pre-COVID-19 and COVID-19 considering all assessed variables (p > 0.05) (Table 1).

**Table 1:** Sociodemographic characteristics according to the time of colorectal cancer diagnosis before and during COVID-19.

Variables	Period		P value
	Pre-COVID-19 (n = 108)	COVID-19 (n = 118)	
**Age at time of diagnosis**			
Median (SD)	60,4 (11,1)	61,4 (12,2)	0,510^t^
Range	(32–84)	(35–84)	
**Sex**			
Female	66 (61,11%)	72 (61,02%)	0,988^q^
Male	42 (38,89%)	46 (38,98%)	
Skin color*			
White	24 (23,53%)	26 (22,22%)	0,821^q^
Black	8 (7,84%)	7 (5,98%)	
Brown	70 (68,63%)	84 (71,79%)	
Education*			
Illiterate	3 (4,29%)	2 (2,11%)	0,498^mc^
Unfinished elementary education	18 (25,71%)	32 (32,68%)	
Finished elementary education	24 (34,29%)	28 (28,47%)	
Finished secondary education	15 (21,43%)	25 (25,32%)	
Finished higher education	10 (14,29%)	8 (8,42%)	
**Place of residence**			
Belo Horizonte	64 (59,26%)	62 (52,54%)	0,378^f^
Others	44 (40,74%)	56 (47,46%)	
**Location of diagnosis**			
Research Institution	87 (80,59%)	100 (84,69%)	0,405^q^
Others	21 (19,41%)	18 (15,31%)	

*: missing information; t: t-test; q: Pearson’s chi-squared test; f: Fisher’s exact test; mc: Chi-squared with Monte Carlo simulation; mw: Mann-Whitney test

Table 2 shows the clinical characteristics between the groups. PS, alcohol consumption, smoking, tumor location, method of diagnosis, type of surgery, protective ostomy, stage, systemic treatment, length of hospitalization, readmission, post-surgical complications, and ICU stay did not present significant variation (p > 0.05).

**Table 2:** Comparison of clinical characteristics between the periods before and during COVID-19

Variables	Period		P value
	Pre-COVID (n = 108)	COVID (n = 118)	
Performance Status*			
0/1	98 (91,6%)	106 (89,8%)	0,838^mc^
2	6 (5,61%)	7 (5,93%)	
3	3 (2,80%)	5 (4,24%)	
Alcoholism*			
Yes	34 (37,36%)	31 (27,68%)	0,187^f^
No	57 (62,64%)	81 (72,32%)	
Smoking*			
Yes	20 (21,74%)	21 (18,75%)	0,723^f^
No	72 (78,26%)	91 (81,25%)	
**Tumor location**			
Ileocecal junction	10 (9,26%)	12 (10,17%)	0,879^mc^
Ascending colon	20 (18,52%)	23 (19,49%)	
Hepatic flexure	5 (4,63%)	2 (1,69%)	
Transverse colon	14 (12,96%)	13 (11,02%)	
Splenic flexure	2 (1,85%)	1 (0,85%)	
Descending colon	7 (6,48%)	9 (7,63%)	
Sigmoide colon	50 (46,30%)	58 (49,15%)	
**Method of diagnosis**			
Emergency surgery	27 (25,0%)	30(25,40%)	0,939 ^q^
Colonoscopy	81 (75,0%)	88 (74,60%)	
**Surgery**			
Radical	60 (55,60%)	73 (61,90%)	0,347^q^
Palliative	48 (44,40%)	45 (38,10%)	
Type of surgery*			
Elective	49 (46,20%)	52 (44,40%)	0,893^q^
Emergency	57 (53,80%)	65 (55,60%)	
**Protective ostomy**			
Yes	52 (48,10%)	44 (37,30%)	0,108^q^
No	56 (51,90%)	74 (62,70%)	
**Stage**			
2	21 (19,4%)	16 (13,56%)	0,061^q^
3	35 (32,4%)	57 (48,31%)	
4	52 (48,2%)	45 (38,14%)	
**Systemic treatment**			
Adjuvant	58 (53,70%)	73 (61,86%)	0,269^f^
Palliative	50 (46,30%)	45 (38,14%)	
**Length of stay at hospital**			
Median (SD)	6,94 (5,26)	7,33 (5,62)	0,715^mw^
Readmission*			
Yes	6 (5,90%)	6 (5,30%)	> 0,999^q^
No	95 (94,10%)	107 (94,70%)	
Post-surgery complication*			
Yes	15 (14,70%)	15 (13,20%)	0,844^q^
No	87 (85,30%)	99 (86,90%)	
ICU stay*			
Yes	43 (46,70%)	46 (44,20%)	> 0,775^q^
No	49 (53,30%)	58 (55,80%)	

*: missing data; q: chi-squared test; f: Fisher’s exact test; mc: Chi-squared with Monte Carlo simulation; mw: Mann-Whitney test; SD: Standard deviation

We assessed the time intervals, in days, between diagnosis, first treatment, request for systemic treatment, and beginning of systemic treatment between groups. Regarding the time between colonoscopy and surgery, the time between surgery and first consultation in clinical oncology, and the time between application for treatment and beginning of systemic treatment, there was significant variation between the assessed groups (p < 0.05) (Table 3).

**Table 3:** Assessment of time intervals in days, from diagnosis to systemic treatment of the study’s sample according to the pandemic period

Variables	Period		P value
	Pre-COVID-19 (n = 108)	During COVID-19 (n = 118)	
**Time between colonoscopy and surgery**			
Median (SD)	59.12 (48.76)	39.92 (44.76)	**0.006** ^**mw**^
**Time between surgery and the date of the first oncological consultation**			
Median (SD)	57.56 (31.92)	45.02 (20.18)	**0.001** ^**mw**^
**Time between colonoscopy and date of first oncological consultation**			
Median (SD)	70 (70)	47 (35)	0.659^mw^
**Time between the date of first oncological consultation and application to systemic treatment**			
Median (SD)	8.01 (15.09)	7.60 (16.33)	0.851^mw^
**Time between the date of request and beginning of systemic treatment**			
Median (SD)	25.45 (16.70)	20.71 (11.35)	**0.020** ^**mw**^
**Total time between diagnostic colonoscopy and beginning of systemic treatment**			
Median (SD)	107 (35)	73 (29)	**< 0.001** ^**mw**^
**Total time between diagnostic surgery and beginning of systemic treatment**			
Median (SD)	91.26 (36.84)	71.87 (23.40)	**< 0.001** ^**mw**^
**Time between the first consultation and beginning of systemic treatment**			
Median (SD)	36.23 (42.18)	29.68 (25.24)	0.115^mw^

SD: Standard deviation. mw: Mann-Whitney test

Table 4 shows the assessment of the characteristics of the studied sample according to staging. No significant association with staging was observed.

**Table 4:** Characteristics of the studied sample according to staging

Variables	Stage			P value
	II	III	IV	
**Sex**				
Female	21 (58.33%)	59 (64.17%)	57 (58.76%)	0.709^q^
Male	15 (41.67%)	33 (35.87%)	40 (41.24%)	
**Marital status**				
Single	14 (42.42%)	27 (31.40%)	31 (36.05%)	0.352 ^mc^
Married	17 (51.52%)	39 (45.35%)	38 (44.19%)	
Widow	1 (3.03%)	10 (11.63%)	12 (13.95%)	
Divorced	1 (3.03%)	10 (11.63%)	5 (5.81%)	
**Skin color**				
White	7 (20.00%)	21 (23.33%)	22 (23.66%)	0.956 ^mc^
Black	3 (8.57%)	5 (5.56%)	7 (7.53%)	
Brown	25 (71.43%)	64 (71.11%)	64 (68.82%)	
**Education**				
Illiterate	1 (3.57%)	1 (1.47%)	3 (4.41%)	0.269^mc^
Unfinished elementary education	4 (14.29%)	23 (33.82%)	23 (33.82%)	
Finished elementary education	12 (42.86%)	21 (30.88%)	18 (26.47%)	
Finished secondary education	6 (21.43%)	14 (20.59%)	20 (29.41%)	
Finished higher education	5 (17.86%)	9 (13.24%)	4 (5.88%)	
**Place of residence**				
Belo Horizonte	22 (61.11%)	50 (54.35%)	53 (54.64%)	0.764^q^
Others	14 (38.89%)	42 (45.65%)	44 (45.36%)	
**Comorbidities**				
Yes	24 (66.67%)	67 (72.83%)	59 (60.82%)	0.217^q^
No	12 (33.33%)	25 (27.17%)	38 (39.18%)	
**Period**				
Pre-COVID-19	20 (55.56%)	35 (38.04%)	52 (53.61%)	0.058^q^
COVID-19	16 (44.44%)	57 (61.96%)	45 (46.39%)	
**Time between diagnostic colonoscopy and beginning of systemic treatment**				
Median (SD)	94 (31)	84 (33)	92 (42)	0.392^kw^
**Time between diagnostic surgery and beginning of systemic treatment**				
Median (SD)	84.42 (24.73)	78.38 (24.20)	83.11 (42.52)	0.256^kw^

mc: Chi-squared with Monte Carlo simulation; q: Chi-square test; kw: Kruskal-Wallis test; SD: Standard deviation

## Discussion

The study’s population presented a similar distribution between men and women, which corroborates the epidemiological data on cancer in Brazil [11]. The average age was over 60 years, similar to the literature that suggests 90% of cases occurred in individuals above 50 years old [12].

The sample of this study consisted of 228 medical records, 108 from the pre-COVID-19 period and 118 from the two-year COVID-19 period, revealing an important decrease in the number of cases treated during the pandemic (45.4% reduction). This finding corroborates the global data that suggested fewer oncological diagnoses. Cancer-tracking programs were put on standby in many countries and, specifically, the tracking of colorectal cancer suffered a harsh downturn [13]. A study in the United Kingdom revealed that the diagnosis of colorectal cancer was reduced by 62% after the beginning of the pandemic [14]. American data indicated a reduction of 45% in the number of colonoscopies done in 2020, in comparison to the annual averages of 2018 and 2019 [15]. A systematic review showed that the volume of colonoscopies of suspected CRC patients has significantly decreased in different countries and at different moments, almost reaching 80% reduction [16]. A Brazilian study revealed a 56.2% reduction in medical consultations in clinical and surgical oncology, regardless of the tumor type [17].

Approximately 75% of diagnoses in both groups were reached through colonoscopy, which is the gold standard for diagnosing and tracking CRC [18].

Although, when assessing surgical treatments, we observed that more than 50% of these procedures were carried out in an emergency. Studies showed that late-stage diseases entail a greater need for emergency procedures due to complications, such as intestinal obstruction or perforation [16, 19].

Regarding the staged assessment, there was no statistical difference between the two assessed periods. On top of that, stages CS III and IV were more frequent in both, the pre-COVID-19, and COVID-19 periods. These data are not corroborated by the literature, which suggested that delays in the trial of cancer during the pandemic resulted in intended late-stage diagnoses, with higher disease severity [20, 21]. Late-stage tumors can also be related to the journey of the oncological patient, from clinical suspicion to diagnosis, which is influenced by many factors, such as difficulty in recognizing symptoms, the difficulty of accessing health services, low educational level, also lack of patient commitment [22]. Moreover, other obstacles hinder the optimal management of cancer patients at SUS regarding the bureaucracies inherent to the public system, culminating in advanced disease [23].

Approximately 40% of surgeries were palliative in both groups, even though, there was no clear benefit of primary tumor resection in the context of metastatic disease, except in situations, such as occlusion, perforation, or bleeding [19].

The literature showed that the pandemic has negatively affected the treatment of CRC patients. A multicentric study carried out in Great Britain verified that, during the COVID-19 pandemic, the treatment of CRC was delayed, which contributed to a 6% increase in the number of deaths related to CRC progression or its complications [24]. According to a meta-analysis by Mazidimoradi et al., the treatment of CRC, including surgery and chemotherapy, was either delayed, interrupted, or suspended during the pandemic [24]. This was also observed in an American study that identified a substantial reduction in the number of colectomies for the treatment of colorectal cancer [25]. These delays were related to work overload, lack of personal protective equipment, lack of workforce, or restricted access to chemotherapy drugs, disrupting the oncological treatment [26]. On the other hand, a previous study performed in the US showed a significant decrease in the number and rate of colorectal cancer diagnoses during the COVID-19 era, with no difference in staging, diagnosis, or time to start treatment [27].

Contrary to what the literature suggests, we observed shorter time intervals during the COVID-19 pandemic when compared with the pre-pandemic period, in terms of: (i) time between diagnosis (colonoscopy or emergency surgery) and beginning of systemic treatment (p < 0.001); (ii) time between colonoscopy and surgery (p = 0.006); (iii) time between surgery and the first oncological consultation (p = 0.001); (iv) time between the date of application for systemic treatment and beginning of treatment (p = 0.020). A possible cause for this finding might be the reduced total number of diagnoses of colorectal cancer during the pandemic, which allowed the health system to be more effective in the treatment of the oncological patient. Cancer, during the pandemic, made the list of priority diseases, changing the patient flow at the institution [28].

Regardless of the period, the time to begin systemic treatment was greater than eight weeks, which disagrees with the guidelines to begin adjuvant treatment [29]. Many studies have shown that a greater delay to begin adjuvant chemotherapy is associated with worse results, both in terms of recurrence-free survival and overall survival in patients with colorectal cancer. The time to begin treatment, as well as the initial stage of the disease, are important prognostic factors [10, 30–33].

In Brazil, one of the strategies to minimize the delay in access to oncological treatment and increase the chance of cure was the sanction of the “Law of the 60 days” (Law 12.732/12), which dictates that a patient diagnosed with cancer has priority to be treated in the Unified Health System (SUS) within 60 days from the time of diagnosis [34]. Although, we observed in this study’s sample that the time to begin treatment exceeded the 60 days required by the law, both before and during the pandemic, a result corroborated by the study of Marcelino et al., carried out in Brazil [23].

Regarding the association of staging with any of the characteristics of the sample, no variations of statistical significance were observed, in any of the variables. This result disagrees with other studies that suggested an increased number of cases with more advanced stages during the pandemic [20, 21]. Our results also disagree with studies that suggested that socioeconomic disparities, such as lack of education and living farther away from health services might be associated with later stages of the disease [35, 36].

Our study presents some limitations as it was based on the retrospective review of medical charts. It relies on the accuracy of written records from a no systematic data registration in medical charts, leading to missing access to important information. Another limitation is the restricted sample size.

## Conclusion

As evidenced in previous literature, the number of colon cancer diagnoses has been significantly reduced during the pandemic period [27]. In disagreement with the available literature [30–33], we observed a reduction in the time between diagnosis and systemic treatment of patients with malignant colon neoplasia during the COVID-19 pandemic. Still, even with this improvement, regardless of the time of diagnosis (before or after the pandemic), the delay to begin treatment remains greater than the eight weeks recommended by current guidelines and exceeds the 60 days, a right of Brazilian patients, which negatively impacts the disease outcome.

Navigating medical care during a pandemic, particularly for vulnerable groups like cancer patients, requires a nuanced and adaptable approach. Cancer patients are typically at higher risk for infections due to their compromised immune system, either from the disease itself or from treatments like chemotherapy. Based on this study’s results, here are some recommendations for the flow of cancer patients during future pandemics: (i) use telemedicine for consultations and follow-ups to reduce the need for patients to come to the hospital and risk exposure; (ii) develop a rigorous triage system to assess the urgency of in-person visits. For example, some check-ups or non-urgent procedures might be postponed, while certain therapies, like cancer surgery, chemotherapy or radiation, may need to continue without delay; (iii) prioritize the approval of cancer patients high complexity procedures by SUS; (iv) prioritize cancer patients for vaccinations (when available and safe for the patient); (v) provide resources for patients to understand how their treatment might be impacted during a pandemic; (vi) allocate dedicated facilities, and treatment areas for cancer patients to minimize their contact with general patient traffic; (vii) collaborate with other cancer treatment centers to share best practices, insights, and strategies for patient care during a pandemic; (viii) develop and regularly update emergency response plans for cancer care during pandemics, taking into account lessons learned from previous health crises. By being proactive and developing a comprehensive strategy that considers both the unique needs of cancer patients and the challenges of a pandemic, healthcare systems can ensure that these vulnerable patients receive the care they need while minimizing their risk of infection.

## Data Availability

All data generated or analyzed during this study are included in this published article.
